# *Obp56h* Modulates Mating Behavior in *Drosophila melanogaster*

**DOI:** 10.1534/g3.116.034595

**Published:** 2016-08-24

**Authors:** John R. Shorter, Lauren M. Dembeck, Logan J. Everett, Tatiana V. Morozova, Gunjan H. Arya, Lavanya Turlapati, Genevieve E. St. Armour, Coby Schal, Trudy F. C. Mackay, Robert R. H. Anholt

**Affiliations:** * Department of Biological Sciences, Program in Genetics and W. M. Keck Center for Behavioral Biology, North Carolina State University, Raleigh, North Carolina 27695; † Department of Entomology and Plant Pathology, Program in Genetics and W. M. Keck Center for Behavioral Biology, North Carolina State University, Raleigh, North Carolina 27695

**Keywords:** odorant binding protein, olfaction, cuticular hydrocarbon, pheromone, 5-tricosene, FlyBook

## Abstract

Social interactions in insects are driven by conspecific chemical signals that are detected via olfactory and gustatory neurons. Odorant binding proteins (Obps) transport volatile odorants to chemosensory receptors, but their effects on behaviors remain poorly characterized. Here, we report that RNAi knockdown of *Obp56h* gene expression in *Drosophila melanogaster* enhances mating behavior by reducing courtship latency. The change in mating behavior that results from inhibition of *Obp56h* expression is accompanied by significant alterations in cuticular hydrocarbon (CHC) composition, including reduction in 5-tricosene (5-T), an inhibitory sex pheromone produced by males that increases copulation latency during courtship. Whole genome RNA sequencing confirms that expression of *Obp56h* is virtually abolished in *Drosophila* heads. Inhibition of *Obp56h* expression also affects expression of other chemoreception genes, including upregulation of *lush* in both sexes and *Obp83ef* in females, and reduction in expression of *Obp19b* and *Or19b* in males. In addition, several genes associated with lipid metabolism, which underlies the production of cuticular hydrocarbons, show altered transcript abundances. Our data show that modulation of mating behavior through reduction of Obp56h is accompanied by altered cuticular hydrocarbon profiles and implicate 5-T as a possible ligand for Obp56h.

Chemical signals are the triggers that guide social interactions in many species (Stowers *et al.* 2013; Liberles 2014). Insects, especially, depend on chemical cues for survival and reproduction. Chemosensation is important for the maintenance of colony structure in social insects (Le Conte and Hefetz 2008; Kocher and Grozinger 2011; Matsuura 2012), and for many insect species is also indispensable for the identification of conspecific mating partners (Ziegler *et al.* 2013; Zhang *et al.* 2015).

*Drosophila melanogaster* provides an excellent model system to investigate the relationship between chemosensation and social behaviors. Males produce 7-tricosene (7-T) and 7-pentacosene (7-P) as primary sex pheromones (Scott 1986), while females produce 7,11-heptacosadiene and 7,11-nonacosadiene (Antony and Jallon 1982; Cobb and Jallon 1990) These cuticular hydrocarbons (CHCs) have been identified as the major contact pheromones in flies essential for mating behavior (Ferveur 2005). In addition, the volatile pheromone, 11-*cis*-vaccenyl acetate, has been implicated in both mating behavior (Kurtovic *et al.* 2007; Ronderos and Smith 2010) and aggression (Wang and Anderson 2010).

Chemosensation in *Drosophila* is mediated via several multigene families of chemoreceptors, including gustatory (Gr) receptors (Scott *et al.* 2001), which evaluate food intake (Scott *et al.* 2001; Marella *et al.* 2006; Weiss *et al.* 2011; Harris *et al.* 2015; Freeman and Dahanukar 2015) and sense carbon dioxide (Kwon *et al.* 2007; Jones *et al.* 2007); classical odorant (Or) receptors, expressed in basiconic and trichoid sensilla (Vosshall *et al.* 1999; Clyne *et al.* 1999), which recognize volatile airborne odorants (Hallem and Carlson 2006); ionotropic receptors (Irs) expressed in coeloconic sensilla, which detect a diverse array of chemicals, including water-soluble compounds (Benton *et al.* 2009); and odorant-binding proteins (Obps). Odorant-binding proteins are soluble proteins secreted into the perilymph that surrounds the dendrites of olfactory sensory neurons. They are the first components of the chemosensory system to interact with airborne chemicals and facilitate the transport of hydrophobic odorants to their membrane-bound receptors (Wojtasek and Leal 1999; Xu *et al.* 2005). In contrast to extensive information about the molecular response profiles of membrane-bound gustatory and olfactory receptors, relatively little functional information is known about Obps.

Obps were first identified as pheromone-binding proteins in the antennae of the male silk moth, *Bombyx mori* (Vogt and Riddiford 1981), where pH-induced conformational changes mediate binding and release of the pheromone (Wojtasek and Leal 1999). In the silk moth group *Antheraea*, two pheromone binding proteins showed preferential binding to specific components of an acetate and aldehyde pheromone blend (Maida *et al.* 2003). In *Drosophila sechellia*, *Obp57d* and *Obp57e* have been implicated in host plant preference in *Drosophila* by affecting the perception of octanoic and hexanoic acids (Matsuo *et al.* 2007; Matsuo 2012).

The *D. melanogaster* genome encodes a family of 51 *Obp* genes (Hekmat-Scafe *et al.* 2002), which has evolved through gene duplication and subsequent subfunctionalization (Vieira *et al.* 2007). Overall, *Obp* genes are structurally diverse, with an average amino acid identity of 10–15%, but can range from 4 to 60% (Zhou 2010). Several *Obp* family members show distinct expression patterns in the antenna (McKenna *et al.* 1994; Shanbhag *et al.* 2001), including *OS-E* (*Obp83b*), *OS-F* (*Obp83a*), *lush* (*Obp76a*), *PBPRP-2* (*Obp19d*), and *PBPRP-5* (*Obp28a*), and eight have been identified in antennal extracts by high-performance liquid chromatography and mass spectrometric analyses (Anholt and Williams 2010). Despite the genetic divergence of *Obp* genes, they are often highly correlated at the level of gene expression (Zhou *et al.* 2009).

Several studies have documented the role of *D. melanogaster* Obps in olfactory behavior. Natural variation in *Obp* genes is associated with variation in olfactory responses to benzaldehyde and acetophenone (Wang *et al.* 2007, 2010; Arya *et al.* 2010). Obp-dependent odorant recognition appears to be combinatorial. Behavioral responses to 16 ecologically relevant odorants tested across 17 knockdown *Obp* RNAi lines revealed that some *Obp* genes had altered behavioral responses to multiple odorants, and some odorants had altered behavioral responses in several *Obp* knockdown lines (Swarup *et al.* 2011). This suggests that individual odorants may interact with multiple Obps, and individual Obps may interact with multiple odorants.

There is increasing evidence that Obps have diverse pleiotropic functions in *D. melanogaster* not limited to olfaction. First, expression of *Obp* genes is not restricted to olfactory tissues; for example, *Obp8a* is expressed in the male accessory gland (Arya *et al.* 2010; St. Pierre *et al.* 2014). Second, expression of *Obp* genes is genetically correlated with expression of other genes that are enriched for diverse gene ontology categories including synaptic transmission, detection of signals regulating tissue development and apoptosis, postmating behavior and oviposition, and nutrient sensing (Arya *et al.* 2010). Third, different physiological and social conditions modulate expression of *Obp* genes (Zhou *et al.* 2009). Fourth, there is direct evidence that *Obp* genes are associated with other traits, including gustatory responses to tastants (Swarup *et al.* 2014) and lifespan (Arya *et al.* 2010).

Here, we show that RNAi-mediated suppression of the expression of *Obp56h* reduces copulation latency, and this behavioral effect is accompanied by an alteration in the composition of CHCs, notably a reduction in the male sex pheromone 5-tricosene (5-T). Using RNA-seq analysis, we observe a number of differentially expressed genes, including *Or19b*, and several genes associated with lipase activity. Our results suggest that *Obp56h* may be associated with pheromone production and affect social recognition via pheromone perception.

## Materials and Methods

### Drosophila stocks and culture

We obtained the *UAS*-RNAi knockdown line targeting *Obp56h*, *Obp56h*^KK111996^, and its co-isogenic control with an empty integration site (*y w*^1118^; *P*{*attP*,*y*^+^,*w*^3′^}) from the Vienna *Drosophila* Stock Center (http://stockcenter.vdrc.at). We obtained two *GAL4* driver strains from the Bloomington *Drosophila* Stock Center (http://flystocks.bio.indiana.edu/): a ubiquitous *tubulin-GAL4* driver line (*y*^1^ *w*∗; *P*{*tubP-GAL4*} *LL7*/*TM3*, *Sb*^1^) and a *Dll-GAL4* driver that has more restricted expression, including in the antennae, labium, legs, and wings (*P*{*w*[*+mW.hs*]=*GawB*}*Dll*^md23^/*CyO*).

All stocks were reared on cornmeal/molasses/agar medium and maintained under standard culture conditions (25°, 12:12 hr light/dark cycle; lights on at 6:00 am) in an environmentally controlled walk-in incubator.

### Behavioral assays

All behavioral assays were performed on F_1_ individuals from crosses of *UAS*-RNAi *Obp* lines to *Tub-GAL4* and/or *Dll-GAL4* lines. F_1_ individuals with *CyO* or *TM3* balancer genotypes were discarded and not assessed. CO_2_ was used as an anesthetic; however, anesthesia exposure was withheld 24 hr prior to behavioral assays. All behavioral assays were conducted in a behavioral chamber (25°) between 8:00 am and 11:00 am. We assessed copulation latency, and phototaxis and geotaxis as additional sensorimotor behaviors. Unless otherwise specified, we used one-way fixed effect ANOVA models of the form *Y* = μ + *G* + ε where *Y* is the phenotype, μ is the overall mean, *G* is the genotype, and ε is the within-genotype residual variance; and/or *t*-tests to evaluate significant differences in behavior among genotypes. All statistical analyses were conducted using SAS (SAS Institute Inc. 2011) software.

#### Copulation latency:

To assess mating behavior, we paired five males and five virgin females aged 3–7 d together in a vial and recorded copulation latency for 30 min. Once a pair engaged in copulation, they were removed from the vial with a mouth aspirator and the remaining flies were observed for the remaining time. We performed at least 40 replicates with five males and five females each per genotype.

#### Geotaxis:

We assessed geotaxis behavior on individual flies by measuring distance traveled upwards following a sudden disturbance. Flies were placed in 25 mm × 150 mm glass vials (Pyrex-Corning flat bottom) with a ruler marking 5-mm increments from 0, indicating the lowest position, to 24, indicating the highest position. Each fly was tapped to the bottom of the vial, and the distance traveled upwards was scored based on the highest point reached in 5 sec. Twenty individual flies were assayed each day for 3 d, creating a total sample size of 60 per sex per genotype.

#### Phototaxis:

We assessed phototaxis behavior using a “countercurrent apparatus” (Benzer 1967). Each replicate per genotype consisted of ∼50 3- to 7-d-old flies of the same sex; we performed three replicates per sex and genotype across 3 d. Flies were dark-adapted for 30 min prior to performing the assay in a dark room. To assess phototaxis, we tapped flies to the bottom of the first start tube and placed the apparatus horizontally with the distal tubes 5 cm away from a 15-W fluorescent light. The flies were given 15 sec to reach the distal tube. We repeated this procedure seven more times, so that flies could choose to go toward the light a maximum of eight times. At the end of each trial, we collected all flies into the start tubes, removed the start tubes from the apparatus and froze them at −80° for ∼30 min before counting the number of flies in each tube. The phototaxis score was analyzed by ANOVA according to the factorial mixed model *Y =* µ *+ G + S + G* × *S + R*(*G* × *S*) *+* ε, where *Y* is the observed value, µ is the overall mean, and *G*, *S*, and *R* denote genotype, sex, and replicate, respectively, and ε is the residual experimental error. Genotype and sex are fixed effects and replicate is random.

### Cuticular hydrocarbon analysis

Cuticular hydrocarbon analysis was performed as described previously (Dembeck *et al.* 2015). We performed two separate experiments, one with *Dll-GAL4* × *UAS-Obp56hRNAi* and *Dll-GAL4* × control F_1_ virgin males, and one with *Tub-GAL4* × *UAS-Obp56hRNAi* and *Tub-GAL4* × control F_1_ virgin males. All males were collected at eclosion and placed in mixed sex groups with five males and five females of the same genotype for 3 d prior to collection for CHC analysis. The flies were separated into three replicate samples per line, with five flies per replicate. To ensure cuticular lipid contamination did not occur, a fresh paper tissue was placed on the carbon dioxide pad and the flies were handled with acetone-washed titanium forceps at each round of sorting. All samples were stored in 2-ml glass auto-injection vials with a Teflon cap and were flash frozen and stored at −30° until cuticular lipid extraction.

Cuticular lipids were extracted from each sample using 200 µl of hexane containing an internal standard (IS, 1 µg *n*-C32) with gentle swirling for 5 min. The flies were briefly extracted a second time with 100 µl of hexane (free of internal standard). After each wash the extract was transferred to a 300 µl conical glass insert. The extract was dried using a gentle stream of high-purity N_2_ and resuspended in 50 µl of hexane. The samples were immediately processed using gas chromatography or stored at 4° (no longer than 1 d) until processing.

The cuticular lipid extracts were analyzed using an Agilent 7890A gas chromatograph with a DB-5 Agilent capillary column (20 m × 0.18 mm × 180 µm) and a flame ionization detector (FID) for quantification. We introduced 1 µl of sample using an Agilent 7683B auto-injector into a 290° inlet operated in splitless mode. The split valve was turned on after 1 min. The oven temperature program was as follows: 50° for 1 min, increased at 20°/min to 150°, and increased at 5°/min to 300° followed by a 10-min hold. Hydrogen was used as the carrier gas at constant flow (average linear velocity = 35 cm/sec) and the FID was set at 300°. Compound identifications were based on a previous GC-MS analysis (Dembeck *et al.* 2015). All chromatograms were analyzed using Agilent ChemStation software. The data were represented as proportions by dividing each peak area by the total sum of all integrated peaks. We analyzed differences in CHCs between *Obp56h*-RNAi knockdown flies and controls using *t*-tests (SAS 9.3). Principal component analysis was conducted on the correlation matrix of the proportions of CHCs quantified in each sample in JMP v.10.

### Gene expression analysis

We used RNA-seq to quantify differences in gene expression in heads and bodies of males and females of *Dll-GAL4* × *UAS-Obp56h* and *Dll-GAL4* × control F_1_ individuals. F_1_ individuals with *CyO* or *TM3* balancer genotypes were discarded. Flies were aged for 5–6 d in a mixed sex environment at a density of ∼20 in a vial. Flies were flash frozen over dry ice between 8:00 am and 11:00 am and 30 heads and bodies per sex and genotype were manually dissected and collected over 3 d in a randomized design, with four biological replicates per sex, genotype, and tissue.

We extracted total RNA with Trizol with the Quick-RNA MiniPrep kit (Zymo Research; R1055). rRNA was depleted using the Ribo-Zero Gold rRNA Removal Kit (Human/Mouse/Rat) (Illumina) with 5 μg total RNA input. Depleted mRNA was fragmented and converted to first strand cDNA using SuperScript III Reverse Transcriptase (Thermo Fisher Scientific). During the synthesis of second strand cDNA, dUTP instead of dTTP was incorporated to label the second strand cDNA (Thermo Fisher Scientific). cDNA from each RNA sample was used to produce barcoded cDNA libraries using NEXTflex DNA Barcodes (Bioo Scientific) with an Illumina TrueSeq compatible protocol. Library size was selected using Agencourt Ampure XP Beads (Beckman Coulter) and centered on 250 bp with average insert size around 130 bp. Second strand DNA was digested with Uracil-DNA glycosylase before amplification to produce directional cDNA libraries. Libraries were quantified using Qubit dsDNA HS Kits (Life Technologies) and Bioanalyzer (Agilent Technologies) to calculate molarity. They were then diluted to equal molarity and requantified, and 32 libraries were pooled. Pooled library samples were quantified to calculate final molarity and finally denatured and diluted to 14 pM. Pooled library samples were clustered on an Illumina cBot and sequenced on an Illumina Hiseq2500 using 125-bp single-read v4 chemistry on each of two lanes.

The quality of the RNA-seq data was assessed using FASTQC (Andrews 2010). Following assessment that the data were of high quality, adapter sequences were trimmed using Cutadapt (Martin 2011). Ribosomal reads were filtered against a database of the most common ribosomal sequences using fast BWA alignment BWA-0.7.10 (Li and Durbin 2009). The remaining reads were aligned to the Dmel_r5.57_FB2014_03 genome and transcriptome using STAR_2.4.1d (Dobin *et al.* 2013). All individual RNA-seq samples had >12 million reads after standard filtering by quality scores and after filtering out of residual rRNA sequences. Read counts for each gene in each sample were computed using HTSeq (v0.6.1p1) (Anders *et al.* 2015). R software was used for further quality assessment and statistical analysis (R-Core-Team 2012). The EDASeq package was used to plot principal components (Risso *et al.* 2011), and one replicate sample (HRNAiF1) was identified as a technical outlier, removed, and the remaining 31 samples were used for analysis. The edgeR package was used to calculate differential expression analysis for pairwise comparisons between the control and RNAi sample for sex and tissue as well as the interaction between genotype and tissue for each sex (Robinson *et al.* 2010). Biological pathway and gene ontology enrichment analyses were performed using DAVID (Huang *et al.* 2009).

### Data availability

RNA-seq data have been deposited in the Gene Expression Omnibus database under accession numbers GSM1959750-GSM1959781.

## Results and Discussion

### RNAi knockdown of Obp56h reduces copulation latency

Previously we investigated the functions of *Drosophila* Obps in olfaction by measuring responses of 17 *Obp*-RNAi lines to 16 odorants (Swarup *et al.* 2011). Quantification of expression of mRNA targets showed a major reduction in the expression of *Obp56h*. Subsequent behavioral studies using these *Obp* RNAi lines indicated that suppression of *Obp56h* expression could influence mating behavior. To further explore the role of Obp56h in mating behavior, we obtained a *UAS*-RNAi knockdown line targeting *Obp56h*, *Obp56h*^KK111996^, and its co-isogenic control with an empty integration site (*y*,*w*^1118^; *P*{*attP*,*y*^+^,*w*^3′^}) from the Vienna *Drosophila* Stock Center (http://stockcenter.vdrc.at). We obtained two *GAL4* driver strains from the Bloomington *Drosophila* Stock Center (http://flystocks.bio.indiana.edu/): a ubiquitously expressed *tubulin-GAL4* driver line (*y*^1^ *w*^∗^; *P*{*tubP-GAL4*} *LL7*/*TM3*, *Sb*^1^) and a *Dll-GAL4* driver, which has more restricted expression, including in the antennae, labium, legs, and wings (*P*{*w*[*+mW.hs*]=*GawB*}*Dll*^md23^/*CyO*).

**Figure 1:**
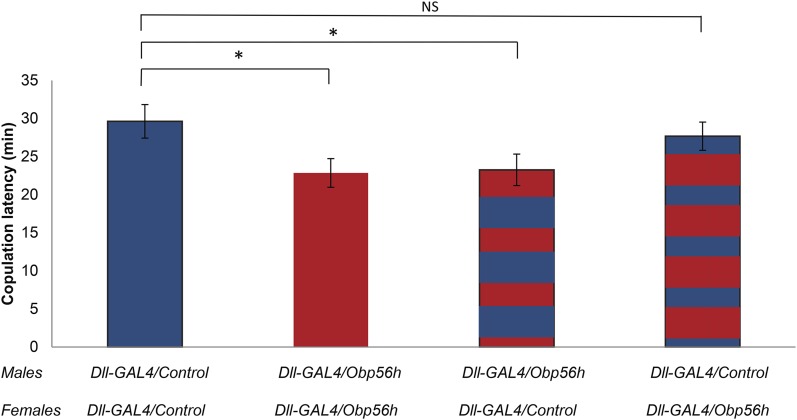
Effect of *Obp56h*-RNAi knockdown on copulation latency. Five males and five virgin females aged 3–7 d were placed together in a vial and copulation latency was recorded for 30 min. We performed at least 40 replicates per genotype (*i.e.*, 200 males and 200 females total per genotype). Red bars denote *Dll-GAL4*/*Obp56h*-RNAi and blue bars denote *Dll-GAL4*/Control F_1_ genotypes; red and blue stacked bars denote pairs of flies with different male and female genotypes. * *P* < 0.05; NS, not significant.

We assessed mating behavior for groups of *Dll-GAL4/*Control males and females (*N* = 42), and for groups of *Dll-GAL4/Obp56h*-RNAi males and females (*N* = 57). We found a significant reduction in copulation latency for the *Obp56h*-RNAi knockdown flies (Figure 1, *t*-test, *t*_98_ = 5.46, *P* = 0.02). This could be due to *Obp56h*-RNAi knockdown males, females, or both sexes. Therefore, we assessed copulation latency for groups of *Dll-GAL4/Obp56h*-RNAi males and *Dll-GAL4/C*ontrol females (*N* = 48) and for groups of *Dll-GAL4/*Control males and *Dll-GAL4/Obp56h*-RNAi females (*N* = 59). We found significantly lower copulation latency for the *Dll-GAL4/Obp56h*-RNAi in males and control females than for the *Dll-GAL4/*Control male and female groups (Figure 1, *t*_89_ = 5.08, *P* = 0.03), but not the control males and *Dll-GAL4/Obp56h*-RNAi females (Figure 1, *t*_100_ = 0.460, *P* = 0.50), indicating that the *Obp56h*-RNAi male genotype was responsible for the reduced copulation latency.

### RNAi knockdown of Obp56h does not have a general effect on sensorimotor behaviors

**Figure 2:**
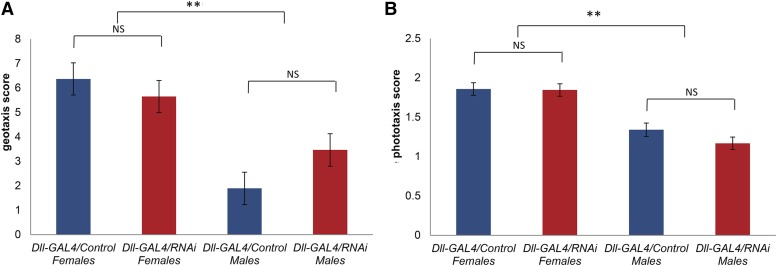
Effects of *Obp56h*-RNAi knockdown on geotaxis and phototaxis. Red bars denote *Dll-GAL4*/*Obp56h*-RNAi and blue bars denote *Dll-GAL4*/Control F_1_ genotypes. (A) Geotaxis. (B) Phototaxis. ** *P* < 0.0001; NS, not significant.

We tested the performance of *Dll-GAL4/*Control and *Dll-GAL4/Obp56h*-RNAi males and females in two behavioral assays that represent sensorimotor responses, geotaxis (*N* = 129–149 per sex), and phototaxis (*N* = 56–67 per sex). We found significant sexual dimorphism for both behaviors (Figure 2), but no significant differences between the two genotypes for geotaxis (Figure 2A, ANOVA *F*_1, 205_ = 7.97, *P* < 0.0001, Genotype *P* = 0.24, Sex *P* < 0.0001, Genotype × Sex *P* = 0.09) or phototaxis (Figure 2B, ANOVA *F*_1, 574_ = 19.49, *P* < 0.0001, Genotype *P* = 0.17, Sex *P* < 0.0001, Genotype × Sex *P* = 0.32). Therefore, the effect of *Obp56h*-RNAi knockdown appeared not to be due to a general effect on locomotion.

### RNAi knockdown of Obp56h alters cuticular hydrocarbon profiles

Many insects, including *Drosophila*, communicate social and sexual information via long-chain cuticular hydrocarbons (CHCs) (Howard and Blomquist 2005; Svetec and Ferveur 2005; Everaerts *et al.* 2010; Ferveur and Cobb 2010). Antiaphrodisiac effects are a common feature of several male-produced pheromones, including the hydrocarbons 5-T and 7-T, and the acetate ester 11-*cis*-vaccenyl acetate (Scott 1986; Ferveur 1997; Canavoso *et al.* 2001; Ng *et al.* 2014).

**Figure 3:**
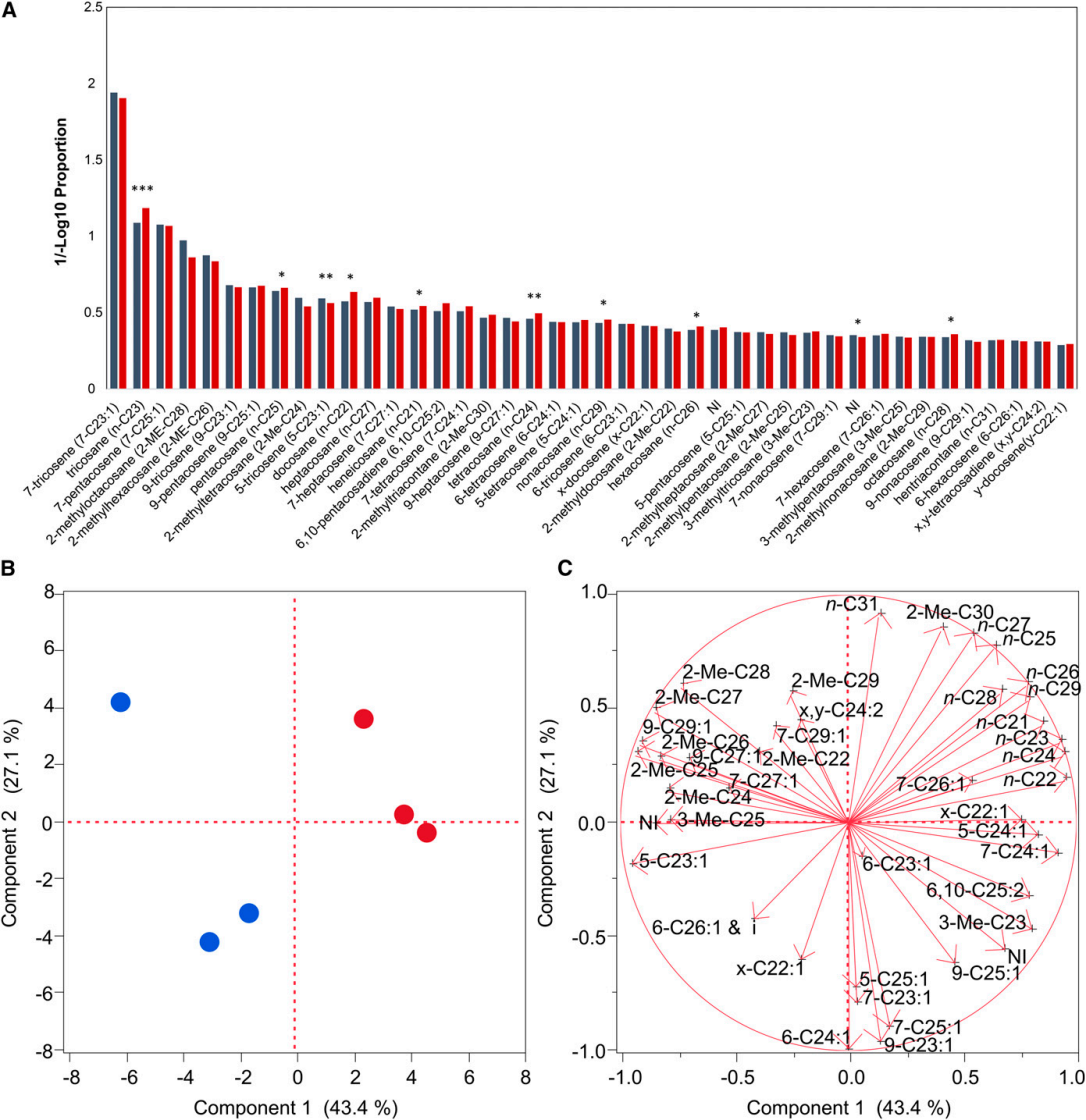
Effects of *Obp*-RNAi knockdown with a *Dll-GAL4* driver on CHC profiles. Cuticular hydrocarbon analysis was performed as described previously (Dembeck *et al.* 2015). (A) Proportion of 42 CHCs in *Dll-GAL4*/*Obp56h*-RNAi (red bars) and *Dll-GAL4*/Control (blue bars) F_1_ males. *** *P* < 0.001, ** *P* < 0.01, * *P* < 0.05. NI, not identified. (B) Principal component biplots for PC1 and PC2 for *Dll-GAL4*/*Obp56h*-RNAi (red circles) and *Dll-GAL4*/Control (blue circles) F_1_ males. The principal components analysis is a linear transformation used to reduce the dimensionality of the multivariate dataset. PC1 captures the variation in the data that can be attributed to genotype (control *vs.* RNAi knockdown) seen by the clustering of the samples into two distinct groups. (C) PC1 and PC2 eigenvectors. The eigenvectors are composed of the weights of each original variable in the linear combinations that define PC1 and PC2. The plot indicates which of the original variables are most strongly correlated with PC1 and PC2. The percent of variance explained by each PC is indicated on the *x*- and *y*-axes of panels (B) and (C).

**Figure 4:**
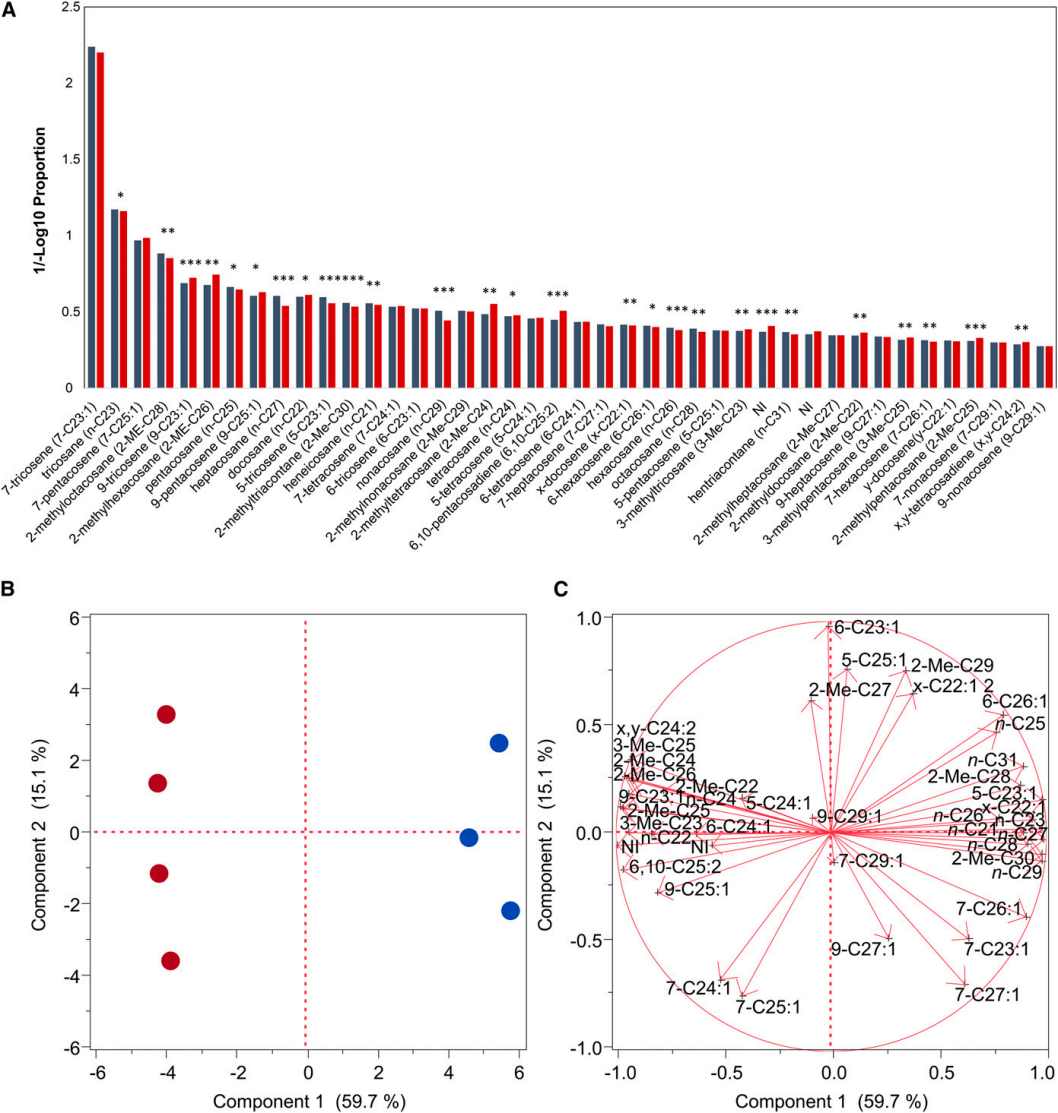
Effects of *Obp*-RNAi knockdown with a *Tub*-GAL4 driver on CHC profiles. Cuticular hydrocarbon analysis was performed as described previously (Dembeck *et al.* 2015). (A) Proportion of 42 CHCs in *Tub-GAL4*/*Obp56h*-RNAi (red bars) and *Tub-GAL4*/Control (blue bars) F_1_ males. *** *P* < 0.001, ** *P* < 0.01, * *P* < 0.05. NI, not identified. (B) Principal component biplots for PC1 and PC2 for *Tub-GAL4*/*Obp56h*-RNAi (red circles) and *Tub-GAL4*/Control (blue circles) F_1_ males. The principal components analysis is a linear transformation used to reduce the dimensionality of the multivariate dataset. PC1 captures the variation in the data that can be attributed to genotype (control *vs.* RNAi knockdown) seen by the clustering of the samples into two distinct groups. (C) PC1 and PC2 eigenvectors. The eigenvectors are composed of the weights of each original variable in the linear combinations that define PC1 and PC2. The plot indicates which of the original variables are most strongly correlated with PC1 and PC2. The percent of variance explained by each PC is indicated on the *x*- and *y*-axes of panels (B) and (C).

To assess whether reduced copulation latency from RNAi knockdown of *Obp56h* could be in part due to differences in chemical communication, we quantified CHC profiles of *Dll-GAL4/Obp56h*-RNAi knockdown and *Dll-GAL4/*Control males. We detected 42 CHCs (Figure 3). The two major male CHC sex pheromones, 7-T and 7-P, were not different between the two genotypes. However, 10 (23.8%) CHCs were significantly altered between the RNAi knockdown and the control (Figure 3). Eight of the ten significantly different CHCs increased relative to the control and are all *n*-alkanes (*n*-C21–*n*-C29, except *n*-C27). Of the two that decreased, one was a minor, unidentified compound, the other was 5-T. 5-T is an inhibitory pheromone, produced primarily in males and only in small quantities in females, that is thought to delay the initiation of courtship in *D. melanogaster* and may serve to decrease the probability of male–male courtship in nature (Ferveur and Sureau 1996; Ferveur 1997; Waterbury *et al.* 1999). 5-T is one of the most volatile *D. melanogaster* CHCs and may be detected through olfaction rather than through contact (Ferveur and Sureau 1996; Ferveur 1997; Waterbury *et al.* 1999). The *Dll-GAL4/Obp56h*-RNAi knockdown males had about 19% less 5-T than the control males. To replicate these observations, we also determined CHC profiles of *Tub-GAL4/Obp56h*-RNAi knockdown and *Tub-GAL4/*Control males (Figure 4). With the ubiquitously expressed tubulin driver line 5-T was also reduced in *Tub-GAL4/Obp56h*-RNAi knockdown males, this time by 32%. The alterations in CHC profiles were similar between the two drivers: nine of the 10 significantly changed CHCs using the *Dll-GAL4* driver also changed when reduction in *Obp56h* expression was driven by *Tub-GAL4*. Thus, interference with chemosensory input through *Obp56h*, and possibly other functions of *Obp56h*, resulted in systemic alterations in CHC biosynthesis.

### Genome-wide changes in gene expression caused by RNAi knockdown of Obp56h

Understanding which genes are coregulated when *Obp56h* expression is reduced by RNAi knockdown can give insights into the biological processes through which *Obp56h* affects mating behavior. Therefore, we performed RNA-seq analysis for *Dll-GAL4/*Control and for *Dll-GAL4/Obp56h*-RNAi males and females, separately for heads and bodies (Supplemental Material, Table S1).

As expected, *Obp56h* expression was significantly reduced in *Dll-GAL4/Obp56h*-RNAi heads in both sexes, with a log-fold change of −3.43 in females (*P* = 1.56 × 10^−29^) and −4.57 in males (*P* = 2.23 × 10^−41^). In addition, *Obp83ef* was up-regulated in *Dll-GAL4/Obp56h*-RNAi female heads and *Obp19b* was down-regulated in *Dll-GAL4/Obp56h*-RNAi male heads. *Or19b* was strongly down-regulated in *Dll-GAL4/Obp56h*-RNAi male heads. Interestingly, *lush* expression was up-regulated in male and female *Dll-GAL4/Obp56h*-RNAi heads. In total, we found 50 (95) differentially expressed transcripts in male (female) heads, 158 (133) differentially expressed transcripts in male (female) bodies, and 54 (170) transcripts with significant genotype × tissue interactions in males (females) at an FDR < 0.05 (Table S2).

Based on the 17,055 FlyBase IDs indicated in Table S1, we performed gene ontology enrichment analyses (Huang *et al.* 2009) for genes with differential expression between the *Obp56h*-RNAi and control genotypes in heads and bodies (Table S3). The most enriched categories in heads and female bodies comprised genes associated with immune/defense responses, which may participate in removal of xenobiotics, including odorants. In addition, and consistent with changes in CHCs, genes associated with the gene ontology terms of lipase, triglyceride lipase activity, and phospholipase activity were also enriched in the bodies of both males and females. Four genes with decreased triglyceride lipase activity and phospholipase activity in male bodies (*CG11598*, *CG6271*, *CG6277*, *CG6283*) are interesting since lipases modify lipids and fatty acids, which are precursors of insect CHCs (Howard and Blomquist 2005; van der Goes van Naters and Carlson 2007). Decreases in expression of genes inferred to have lipase activity could provide a mechanistic basis for the altered CHC profiles.

The promoter of *Obp56h* expresses *lacZ* in approximately five sensilla on each third antennal segment, in the pharyngeal organs and in the dorsal organ, the terminal organ, and the ventral pits of the third instar larvae (Galindo and Smith 2001). This Obp, therefore, may function in both olfactory and gustatory systems. It is of interest that expression of *Or19b* is down-regulated in *Obp56h*-RNAi male heads, especially since *Or19b* is expressed in trichoid sensilla (Couto *et al.* 2005), which appear specialized for the detection of pheromones (Ha and Smith 2006; Ronderos and Smith 2010). *Or19b* is thus a plausible candidate receptor for 5-T or another unknown *Obp56h* ligand.

## Supplementary Material

Supplemental Material
