# Supplementary material for: Obp56h Modulates Mating Behavior in Drosophila melanogaster
Source: G3 (Bethesda). 2016 Aug 24;6(10):3335–42. doi: 10.1534/g3.116.034595 (PMC5068952; doi:10.1534/g3.116.034595)
Supplement: Supplemental Material [file supp_6_10_3335__index.html]

Obp56h Modulates Mating Behavior in Drosophila melanogaster — Supplemental Material 

# *Obp56h* Modulates Mating Behavior in *Drosophila melanogaster*

## Supplemental Material for Shorter *et al.*, 2016

**Files in this Data Supplement:**

- Table S1 - Results of three-way factorial analyses of variance of gene expression. (.xlsx, 7 MB)
- Table S2 - Significant (FDR < 0.05) differentially expressed genes between *Obp56h* RNAi knockdown and control genotypes, from analyses of variance for males and females separately. (.xlsx, 94 KB)
- Table S3 - GO enrichment analyses for significant (FDR < 0.05) differentially expressed genes in *Opb56h* RNAi knockdown and control genotypes. (.xlsx, 14 KB)
